# Inhibition of tumor suppressor p73 by nerve growth factor receptor via chaperone-mediated autophagy

**DOI:** 10.1093/jmcb/mjaa017

**Published:** 2020-04-13

**Authors:** Daniel Nguyen, Kun Yang, Lucia Chiao, Yun Deng, Xiang Zhou, Zhen Zhang, Shelya X Zeng, Hua Lu

**Affiliations:** 1 Department of Biochemistry and Molecular Biology, Tulane Cancer Center, Tulane University School of Medicine, New Orleans, LA 70112, USA; 2 Department of Radiation Oncology, Shanghai Cancer Center, Department of Oncology, Shanghai Medical School, Fudan University, Shanghai 200032, China; 3 Verna and Marrs McLean Department of Biochemistry and Molecular Biology, Baylor College of Medicine, Houston, TX 77030, USA; 4 Department of Radiation Oncology, Shanghai Cancer Center, Fudan University, Shanghai 200032, China; 5 Institute of Biomedical Sciences, Shanghai Cancer Center, Fudan University, Shanghai 200032, China

**Keywords:** p73, NGFR, chaperone-mediated autophagy, Lamp2a, HSPA8

## Abstract

The tumor suppressr p73 is a homolog of p53 and is capable of inducing cell cycle arrest and apoptosis. Here, we identify nerve growth factor receptor (NGFR, p75NTR, or CD271) as a novel negative p73 regulator. p73 activates NGFR transcription, which, in turn, promotes p73 degradation in a negative feedback loop. NGFR directly binds to p73 central DNA-binding domain and suppresses p73 transcriptional activity as well as p73-mediated apoptosis in cancer cells. Surprisingly, we uncover a previously unknown mechanism of NGFR-facilitated p73 degradation through the chaperone-mediated autophagy (CMA) pathway. Collectively, our studies demonstrate a new oncogenic function for NGFR in inactivating p73 activity by promoting its degradation through the CMA.

## Introduction

p73, along with p53 and p63, belongs to the p53 family of tumor suppressors (Kaghad et al., 1997). p53 is widely regarded as the guardian of the genome as it is capable of responding to a variety of cellular stressors, including oxidative stress, oncogene activation, ribosomal dysfunction, and DNA damage, by inducing protective mechanisms such as cell cycle arrest, apoptosis, DNA repair, and inhibition of angiogenesis and metastasis (Sengupta and Harris, 2005; Levine and Oren, 2009; Zhang and Lu, 2009; Bieging and Attardi, 2012; Meek, 2015). p73 has also been shown to play a role in tumor suppression (Flores et al., 2005; Tomasini et al., 2008, 2009). Like p53, p73 functions as a transcription factor that forms tetramers and binds to target DNA, transcribing genes involved in numerous biological activities, and shares significant homology to p53 in domain structure, as there is 22% homology in the N-terminal transactivation domain, 63% in the central DNA-binding domain, and 38% in the C-terminal oliogomerization domain (Scoumanne et al., 2005). This structural homology, particularly in the DNA-binding domain, accounts for the observed functional redundancy, as p73 is capable of transcribing canonical p53 targets, such as p21, MDM2, Puma, and Bax, and can therefore likewise induce cell cycle arrest, apoptosis, and senescence (Harms et al., 2004). p73 exists as multiple isoforms which can be divided functionally into full-length or TA-isoforms that contain the transactivating domain and differ only by the C-terminus via alternative gene-splicing, and the ΔNp73 isoform which, due to distinct promoter usage, does not contain the transactivating domain. While little is known concerning the phenotypic differences of different TAp73 isoforms (named p73α, p73β, p73γ, etc.), they by virtue of the transactivating domain transcribe canonical targets and are thereby associated with tumor suppression. On the other hand, ΔNp73 does not transcribe such targets and in fact has been found to function as an oncoprotein (Harms et al., 2004; Scoumanne et al., 2005).

Because of p73 critical role in tumor suppression, understanding its mechanisms of regulation is vital. Although MDM2 is the primary E3 ubiquitin ligase for p53, it does not target p73 for proteosomal degradation (Haupt et al., 1997; Marine and Lozano, 2010). MDM2 can, however, still interact with p73 and suppresses p73 transcriptional activity by competing with co-activator p300/CBP without affecting p73 stability (Zeng et al., 1999). Instead, the primary E3 ligase that has been associated with p73 is ITCH, a HECT (homologous to E6-AP carboxyl terminus)-containing Nedd4-like ubiquitin ligase, which binds to the proline-rich domains in the C-terminus and promotes proteosomal degradation (Rossi et al., 2005). Other E3 ligases have also been identified that regulate p73 stability, such as triparite motif protein 32 (TRIM32) is an E3 ligase that promotes p73 ubiquitylation and degradation, while itself being a transcriptional target of p73, mirroring the negative feedback loop observed between p53 and MDM2 (Gonzalez-Cano et al., 2013). Non-canonical degradation pathways have also been identified. The enzyme NAD(P)H dehydrogenase quinone oxidoreductase 1 (NQO1) has been shown to physically interact with p73 in an NADH-dependent manner and protects them from ubiquitin-independent proteolysis by the 20S proteasome (Asher et al., 2005). Another ubiquitin-independent mechanism involves the U-box-E3/E4 ubiquitin ligase UFD2a, which binds to p73 at its C-terminal SAM domain and promotes its proteosomal turnover without affecting its ubiquitination levels (Hosoda et al., 2005). Furthermore, the transcription factor c-Jun has been shown to specifically target ΔNp73 for degradation via the polyamine-induced antizyme (Az) pathway (Dulloo et al., 2010).

Our previous studies have shown that nerve growth factor receptor (NGFR), a single transmembrane receptor involved in nervous system development, regulates p53 activity as a negative feedback regulator by enhancing its ubiquitination by MDM2 (Zhou et al., 2016b). NGFR is found to be highly expressed in a variety of cancers, and its association with cancer cell survival, invasive potential, and propagation stem cell-like phenotype may be linked to p53 inactivation (Descamps et al., 2001; Barker, 2004; Soland et al., 2008; Truzzi et al., 2008; Boiko et al., 2010; Civenni et al., 2011; Kim et al., 2012). Our study also suggested that NGFR plays a p53-independent oncogenic role (Zhou et al., 2016b). Although NGFR was previously shown to play a role in brain neuron development in response to p73 (Niklison-Chirou et al., 2013), it remains unclear how NGFR executes p53-independent oncogenic activity and whether this activity engages p73 or not. Here, we identify a novel mechanism of NGFR-dependent p73 regulation through the chaperone-mediated autophagy (CMA) pathway. CMA differs from macroautophagy, in which CMA is a selective process in which substrates bearing a ‘KFERQ’-like motif are recognized by HSPA8 and delivered directly to the lysosomes via the receptor LAMP2a, and transported into the lysosomal lumen for degradation (Kiffin et al., 2004; Cuervo and Wong, 2014). CMA activity has been found to be enhanced in cancers and is required for tumor growth and survival through mechanisms such as protection against oxidative stress, targeted removal of tumor suppressors, and maintenance of metabolic switch favoring cell growth (Kon et al., 2011; Lv et al., 2011; Saha, 2012; Quintavalle et al., 2014; Zhang et al., 2014; Zhou et al., 2016a). As detailed below, our studies elucidate a novel regulatory mechanism of p73 tumor suppressive activity, by which NGFR promotes p73 degradation through the CMA activity.

## Results

### NGFR and p73 levels are inversely correlated with each other in cancer cells

**Figure 1:**
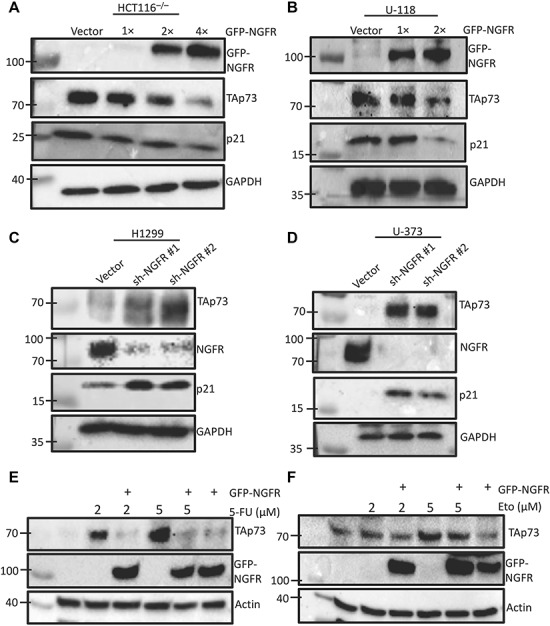
NGFR and TAp73 levels are inversely correlated with each other. (**A** and **B**) Ectopic NGFR expression reduces TAp73 expression and activity. HCT116^p53−/−^ (**A**) and U-118 (**B**) cells were transfected with different doses of GFP-NGFR for 48 h followed by immunoblotting (IB) with the indicated antibodies. (**C** and **D**) NGFR knockdown induces TAp73 expression and activity. H1299 (**C**) and U-373 (**D**) cells were transduced with two lentiviral-based plasmids expressing shRNA targeting different sequences against NGFR or control shRNA for 48 h followed by IB with the indicated antibodies. (**E** and **F**) NGFR inhibits TAp73 activation by 5-FU or Eto. HCT116^p53−/−^ cells were transfected with GFP-NGFR for 30 h and then treated with 5-FU (**E**) or Eto (**F**) for 18 h prior to lysis and IB with the indicated antibodies.

To test whether NGFR expression affects endogenous p73 levels in cancer cells, we first overexpressed NGFR in HCT116^p53−/−^ and mutant p53-containing glioblastoma U-118 cells and detected the protein levels of NGFR and p73 by western blot (WB) analysis. The antibody used in endogenous p73 detection recognizes the N-terminus of p73 and therefore recognizes TAp73 but excludes ΔNp73. As a result, ectopic NGFR at different doses reduced the TAp73 and p21 protein levels in the cells tested (Figure 1A and B) without affecting their RNA levels (Supplementary Figure S1A). Inversely, knockdown of NGFR with two different shRNAs strikingly elevated the levels of endogenous TAp73 and p21 in H1299 and U-373, another glioblastoma cell line with mutated p53 (Figure 1C and D). To exclude the possibility of off-target effects of the two independent shRNA sequences used against NGFR, we performed a rescue experiment by introducing ectopic NGFR expression following the transfection of shRNA sequences against NGFR and observed a rescue of shNGFR-mediated induction of TAp73 (Supplementary Figure S1B). To further elucidate the functional relationship between p73 expression and NGFR, we checked whether NGFR affects TAp73 protein levels in HCT116^p53−/−^ treated with chemotherapeutic reagents known to induce TAp73. Indeed, TAp73 levels induced by 5-fluorouracil (5-FU) and etoposide (Eto) at different doses were markedly reduced by ectopic NGFR (Figure 1E and F). These results indicate that not only NGFR expression is inversely correlated with TAp73 levels in cancer cells, but NGFR can also suppress TAp73 activation by chemotherapeutic reagents.

### p73 transcriptionally activates NGFR expression in human cancer cells

Our previous studies have identified NGFR as a *bona fide* transcriptional target of p53 (Zhou et al., 2016b), and it was also previously shown to have a similar role to that of p73 in mouse neuron morphology and function as a p73 target (Niklison-Chirou et al., 2013). Here, we sought to confirm whether human NGFR is also a direct transcriptional target of human p73. To do so, we overexpressed p73β in human p53-null colon cancer HCT116^p53−/−^ and lung cancer H1299 cell lines and performed RT-qPCR analysis for NGFR mRNA expression with p53 and its target p21 as positive controls. As expected, the expression of NGFR mRNA was consistently elevated in both cell lines tested (Supplementary Figure S2A and B). Furthermore, ectopic expression of p73β also induced NGFR at the protein level (Supplementary Figure S2C). To supplement these findings, we performed a luciferase reporter assay in H1299 cells using two potential response elements in the NGFR promoter identified by bioinformatics analysis in our previous study, RE1 and RE2, and discovered that p73β induced luciferase activity through both RE1 and RE2, indicating that TAp73 is associated with both response elements (Supplementary Figure S2D). Interestingly, p53 was found to only induce luciferase reporter activity through RE1 (Zhou et al., 2016b). While the tumor suppressive activity of full-length or TAp73 has been well established, the ΔNp73 isoform, which lacks the transcriptional active domain, has been observed to be overexpressed in some cancer cells and may instead play an oncogenic role. To determine whether ΔNp73 can also induce NGFR expression, we overexpressed either full-length p73 or ΔNp73 in H1299 cells and found that only full-length p73 can induce NGFR expression (Supplementary Figure S2E). These results confirm that NGFR is a direct transcriptional target of TAp73 in human cancer cells as well.

### NGFR reduces p73 transcriptional activity

**Figure 2:**
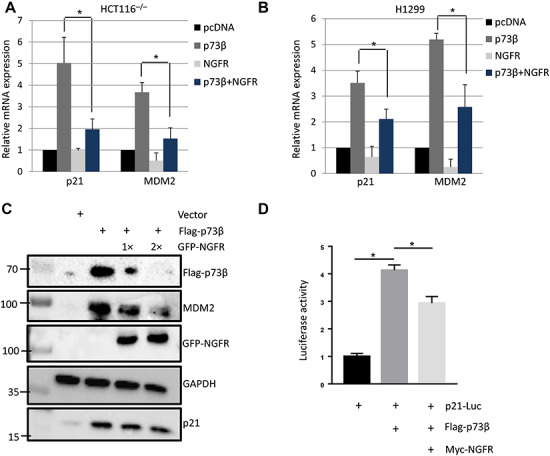
NGFR reduces TAp73 transcriptional activity. (**A** and **B**) Ectopic NGFR expression reduces p73β-mediated transcriptional activity. HCT116^p53−/−^ (**A**) and H1299 (**B**) cells were transfected with the indicated plasmids for 30 h followed by q-PCR analysis for p21 and MDM2. Three biological replicates were used for *P*-value, *P < 0.05. (**C**) HCT116^p53−/−^ cells were transfected with the indicated plasmids for 48 h followed by IB with the indicated antibodies. (**D**) Ectopic NGFR represses the luciferase activity of p21-Luc driven by p73. H1299 cells were transfected with the indicated plasmids, p21-Luc (WWP-Luc) and Renilla-Luc (pRL-TK), for 48 h, followed by the measurement of firefly luciferase activity and normalization by a factor of Renilla luciferase activity in the same assay. Three biological replicates were used for *P*-value, *P < 0.05.

Next, we tested whether NGFR affects TAp73 transcriptional activity. First, we ectopically expressed p73β in p53-null cell lines with or without co-expression with NGFR and analyzed the expression of p73-target genes p21 and MDM2 by RT-qPCR. Ectopic NGFR alone served as a negative control. As expected, p73β overexpression induced p21 and MDM2 mRNA levels, but when cells were co-transfected with NGFR, the induction of p21 and MDM2 was significantly reduced (Figure 2A and B). This repression was also true at the protein level, as ectopic expression of p73β induced p21 and MDM2 protein expression, but co-transfection with NGFR at different doses reduced these protein levels and even reduced exogenous p73β itself (Figure 2C). To further verify NGFR’s suppression of TAp73 transcriptional activity, we conducted a luciferase reporter assay. As shown in Figure 2D, p73β-induced luciferase activity driven by the p21 promoter was significantly reduced in H1299 cells when p73β was co-transfected with NGFR. Collectively, these results demonstrate that NGFR reduces TAp73 transcriptional activity.

### NGFR interacts with p73

**Figure 3:**
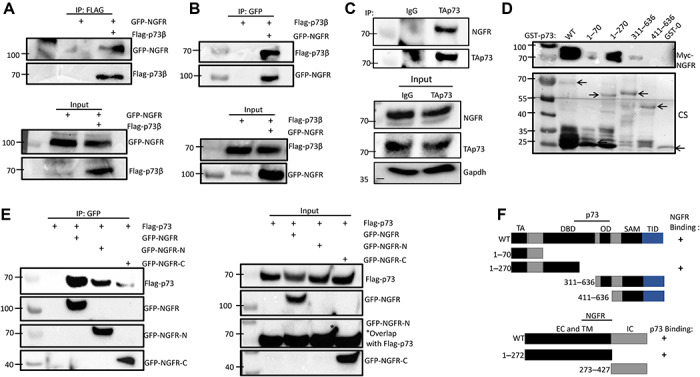
NGFR interacts with p73. (**A** and **B**) NGFR interacts with p73. H1299 cells were transfected with plasmids encoding Flag-p73β, GFP-NGFR, or both, followed by co-IP and IB with the indicated antibodies. (**C**) NGFR and TAp73 associate endogenously. SK-MEL-147 cells were treated with 2 μM 5-FU for 16 h to induce p73 expression, and lysates were immunoprecipitated with either TAp73 or IgG control, followed by IB with indicated antibodies. (**D**) Mapping the NGFR-binding domain on p73 by GST-pulldown assay. GST-tagged p73 fragments corresponding to full-length p73 (WT), aa 1–70, aa 1–270, aa 363–636, and aa 411–636 along with GST protein alone (GST-0) were incubated with cell lysates overexpressing Myc-NGFR, and bound proteins were detected by IB using anti-NGFR antibody or Coomassie staining (CS). (**E**) Mapping the p73-binding domain on NGFR. H1299 cells transfected with Flag-p73 were co-transfected with GFP-NGFR, GFP-NGFR-N (aa 1–272), or GFP-NGFR-C (aa 272–427), followed by co-IP and IB with the indicated antibodies. (**F**) A schematic of NGFR-binding region on p73 and p73-binding region on NGFR.

Next, we wanted to determine how NGFR reduces TAp73 level and activity. First, we tested whether NGFR interacts with p73 by performing a set of reciprocal co-immunoprecipitation (co-IP) coupled with WB assays. Indeed, ectopic NGFR was able to bind to ectopic p73β, and *vice versa* (Figure 3A and B). To ascertain whether endogenous TAp73 and NGFR associate, we utilized the melanoma cell line SK-MEL-147 and immunoprecipitated NGFR with p73 antibody, confirming endogenous interaction (Figure 3C). We then mapped their binding domains by conducting a set of GST-pulldown and co-IP assays. We first mapped the NGFR-binding domain on p73 by employing a GST-pulldown assay. GST-tagged p73 fusion proteins containing full-length p73 and fragments corresponding to amino acids (aa) 1–70 (N-terminal transactivation domain), aa 1–270 (DNA-binding domain), aa 311–636 (C-terminus, including oligomerization domain), and aa 401–636 (C-terminus) were purified from *Escherichia coli*. Interestingly, NGFR was only able to bind to full-length p73 and the fragment containing the DNA-binding domain (aa 1–270), indicating that NGFR binds to p73 central DNA-binding domain (Figure 3D). We also mapped the p73-binding domain on NGFR by co-IP with either full-length NGFR or fragments corresponding to NGFR N-terminal, extracellular and transmembrane domain (aa 1–272) or its C-terminal, intracellular domain (aa 273–427). Like p53 (Zhou et al., 2016b), p73 was able to bind to NGFR N-terminal domain with much less signals in the C-terminal binding (Figure 3E). These results show that while NGFR binds to the central DNA-binding domain on p73, p73 binds to the extracellular and transmembrane N-terminal domain of NGFR (Figure 3F).

### NGFR does not interact with p63

Since p63 also shares significant homology with p53 and p73, particularly in the DNA-binding domain, we also checked to see whether p63 and NGFR also have a functional relationship. Remarkably, ectopic p63 expression was also able to transcriptionally upregulate NGFR in p53-null cells (Supplementary Figure S3A and B). However, reciprocal co-IP–WB assays showed that p63 and NGFR do not interact, suggesting that NGFR might not play a direct role in the regulation of p63 (Supplementary Figure S3C and D).

### NGFR downregulates p73 expression and inhibits p73 activity independently of MDM2

**Figure 4:**
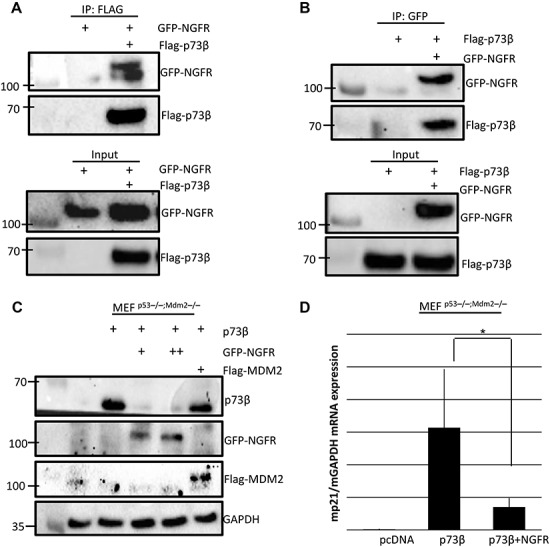
NGFR downregulates p73 expression and inhibits p73 activity independently of MDM2. (**A** and **B**) NGFR interacts with TAp73 in the absence of MDM2. MEF^p53−/−;MDM2−/−^ cells were transfected with plasmids encoding Flag-p73β, GFP-NGFR, or both, followed by co-IP and IB with the indicated antibodies. (**C**) NGFR downregulates TAp73 protein expression even in the absence of MDM2. MEF^p53−/−;MDM2−/−^ cells were transfected with the indicated combinations of plasmids followed by IB. (**D**) NGFR represses p73-induced target gene expression independently of MDM2. MEF^p53−/−;MDM2−/−^ cells were transfected with p73β alone or co-transfected with NGFR for 30 h followed by q-PCR analysis of mouse p21 mRNA levels. Three biological replicates were used for *P*-value, *P < 0.05.

The discovery that NGFR binds to p73 DNA-binding domain, and not its N-terminal domain, suggests that NGFR may inhibit TAp73 transcriptional activity independently of MDM2. To test this hypothesis, we first evinced whether TAp73 and NGFR can interact with each other without the presence of MDM2 by expressing ectopic p73β and NGFR in MEF^p53−/−;MDM2−/−^ cells and performing a reciprocal co-IP–WB assay. Interestingly, p73β and NGFR were still able to interact in these MEF^p53−/−;MDM2−/−^ cells (Figure 4A and B). Next, we determined whether NGFR can downregulate p73β expression independently of MDM2 by co-transfecting p73β with NGFR at different doses in MEF^p53−/−;MDM2−/−^ cells. Interestingly, NGFR was still able to downregulate exogenous p73β, while re-expressing MDM2 had no apparent effect on p73β level (Figure 4C). These results differ from our previous study regarding p53, as NGFR was unable to downregulate p53 expression without MDM2 (Zhou et al., 2016b), suggesting that NGFR regulates TAp73 by a different mechanism. This result is perhaps not all surprising, given that MDM2 was known to not promote p73 degradation (Zeng et al., 1999). However, MDM2 can suppress p73 transcriptional activity, so we determined whether the NGFR inhibition of p73 activity occurs in the absence of MDM2. Indeed, NGFR repressed ectopic-p73β-induced expression of p21 in MEF^p53−/−;MDM2−/−^ cells (Figure 4D). Again, considering our previous finding that NGFR interacts with p73 central DNA-binding domain (Figure 3C), this finding is consistent with literature demonstrating that the MDM2 suppression of p73 activity occurs by binding to p73 N-terminal domain (Zeng et al., 1999). Altogether, these results demonstrate that NGFR both downregulates p73 expression and represses p73 transcriptional activity independently of MDM2.

### NGFR degrades p73 through the CMA pathway

**Figure 5:**
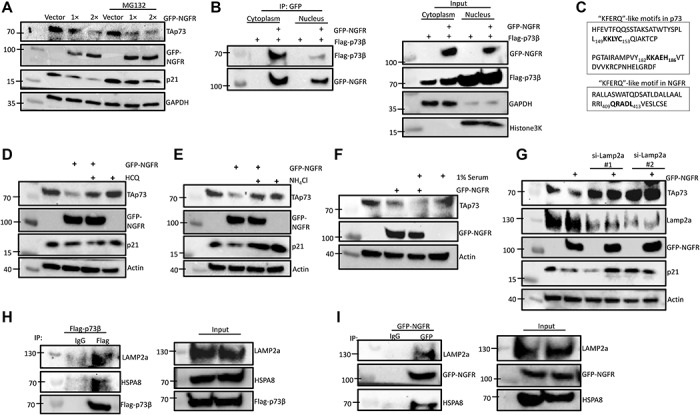
NGFR degrades p73 through the CMA pathway. (**A**) NGFR downregulates p73 independent of proteasome. HCT116^p53−/−^ cells were transfected with different doses of GFP-NGFR for 36 h, followed by treatment with MG132 or DMSO control for 6 h and IB with the indicated antibodies. (**B**) NGFR interacts with TAp73 in the cytoplasm. HCT116^p53−/−^ cells were transfected with Flag-p73β alone or co-transfected with GFP-NGFR for 30 h, followed by cellular fractionation to separate the cytoplasmic and nuclear fractions for co-IP and IB. GAPDH and Histone3K served as controls for cytoplasmic and nuclear fractions, respectively. (**C**) KFERQ-like motifs identified in the amino acid sequences of p73 and NGFR. (**D** and **E**) Inhibition of lysosome function rescues NGFR-mediated downregulation of TAp73. HCT116^p53−/−^ cells were transfected with GFP-NGFR or not for 24 h, followed by treatment with either HCQ (**D**) or NH_4_Cl (**E**) for 18 h before IB with the indicated antibodies. (**F**) Serum starvation enhances NGFR-mediated downregulation of TAp73. HCT116^p53−/−^ cells were transfected with GFP-NGFR or not for 24 h, followed by the removal of medium and replacement with 1% serum for 24 h before IB with the indicated antibodies. (**G**) Knockdown of LAMP2a rescues NGFR-mediated downregulation of TAp73. HCT116^p53−/−^ cells were transfected with two siRNAs targeting different sequences against LAMP2a or screener control for 36 h, followed by transfection with GFP-NGFR and IB with the indicated antibodies. (**H** and **I**) NGFR and TAp73 interact with HSPA8 and LAMP2a. HCT116^p53−/−^ cells were transfected with Flag-p73β (**H**) or GFP-NGFR (**I**), followed by co-IP and IB with the indicated antibodies.

Next, we sought to determine the mechanism by which NGFR promotes the downregulation of p73. Since a major pathway that regulates p73 flux is ubiquitination and subsequent degradation by the proteasome, we first examined whether the inhibition of proteasome activity with MG132 could rescue NGFR-mediated downregulation of TAp73 (Oberst et al., 2005). Surprisingly, in HCT116^p53−/−^ cells transfected with NGFR at different doses, treatment with MG132 did not affect the reduction of TAp73 levels, indicating that NGFR-mediated downregulation of TAp73 does not occur through the proteasome (Figure 5A). MG132’s effect was confirmed by observing p21 levels, which clearly demonstrated increased stability upon treatment. To explore non-canonical mechanisms of p73 regulation by NGFR, we conducted cellular fractionation to separate cytoplasmic and nuclear compartments in cells ectopically expressing p73β and NGFR. Then, we performed co-IP to determine whether the NGFR–p73 interaction occurs primarily in the cytoplasm or the nucleus. To our astonishment, NGFR–p73 interaction was observed predominantly in the cytoplasm, despite the majority of ectopic p73β localizing to the nucleus (Figure 5B). An alternative protein degradation pathway that occurs in the cytoplasm is CMA, in which cytosolic substrate proteins are transported directly to the lysosome for degradation. The chaperone heat shock 70 kDa protein 8 (HSPA8 or HSC70) recognizes proteins bearing a KFERQ-like motif and delivers the substrate to the lysosome via integral membrane protein lysosome-associated membrane protein type 2a (LAMP2a) (Cuervo and Wong, 2014). By examining the amino acid sequences of p73 and NGFR, we have identified two putative pentapeptide sequences (_149_KKLYC_153_ and _182_KKAEH_186_) in p73 and one sequence in NGFR (_409_QRADL_413_) that are consistent with the HSPA8 recognition motif (Figure 5C). To determine whether NGFR promotes p73 degradation through lysosomes, we treated cells transfected with ectopic NGFR with hydroxychloroquine (HCQ) or ammonium chloride (NH_4_Cl), which inhibit lysosome function by raising lysosomal pH and impairing pH-sensitive proteases. As a result, blocking lysosome activity rescued NGFR-mediated downregulation of TAp73 (Figure 5D and E). Treatment with HCQ or NH_4_Cl alone did not alter TAp73 levels. Since prolonged serum starvation can activate the CMA, we assessed the effect of serum deprivation on cells transfected with ectopic NGFR and found that growth in 1% serum as opposed to 10% serum enhanced NGFR-mediated downregulation of TAp73 (Figure 5F; Cuervo et al., 1995). Serum starvation alone did not alter TAp73 levels. To confirm that NGFR mediated p73 degradation through the CMA, we examined the effect of knocking down LAMP2a, the key receptor on the lysosome surface which arbitrates the rate limiting step of the CMA by binding to substrates (Bandyopadhyay et al., 2010). Indeed, knockdown of LAMP2a expression with si-RNA completely rescued NGFR-mediated downregulation of TAp73 (Figure 5G). Finally, we performed co-IP experiments to determine whether p73 and NGFR interact with HSPA8 and LAMP2a. Ectopic expression of Flag-p73β in HCT116^p53−/−^ cells, followed by co-IP with anti-Flag antibody and WB analysis with HSPA8 and LAMP2a, showed that p73β co-immunoprecipitated with endogenous HSPA8 and LAMP2a (Figure 5H). Furthermore, GST fusion protein association assay was performed and showed that GST-tagged p73α fusion proteins containing full-length p73 or fragments corresponding to aa 1–70 (N-terminal transactivation domain) and aa 1–270 (DNA-binding domain) co-immunoprecipitated with endogenous HSPA8 and LAMP2A (Supplementary Figure S4). Similarly, ectopic expression of GFP-NGFR, followed by IP with anti-GFP and IB with HSPA8 and LAMP2a, showed that NGFR also co-immunoprecipitated with endogenous HSPA8 and LAMP2a (Figure 5I). Altogether, these data identify the CMA pathway as the mechanism by which NGFR promotes p73 degradation.

### NGFR attenuates p73 suppression of cancer cell proliferation and growth

**Figure 6:**
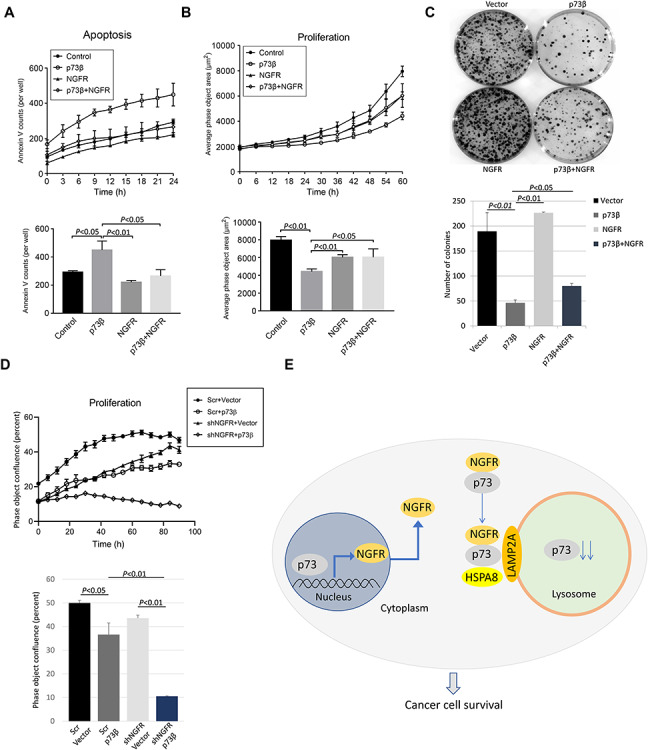
NGFR attenuates p73-mediated tumor suppressive activity. (**A** and **B**) H1299 cells transfected with the indicated plasmids were examined for cell apoptosis (**A**) and proliferation (**B**) by IncuCyte^®^ S3 Live Cell Analysis System. Real-time quantification of time-lapse curves depicting normalized green object count or average phase object area per well at each time point. Accompanying histogram depicts the data at 24 h (apoptosis) and 60 h (proliferation). Three biological replicates were used for *P*-value, *P < 0.05. (**C**) NGFR reduces p73-mediated inhibition of clonogenicity. H1299 cells were transfected with the indicated plasmids and seeded into 60-mm plates the next day. Colonies were fixed by methanol and stained with crystal violet solution. Quantification of colonies is shown below. Two biological replicates were used for *P*-value, *P < 0.05. (**D**) Cells with shRNA-mediated stable knockdown of NGFR are more susceptible to arrest in proliferation by TAp73. SK-MEL-147 cells with stable expression of either shRNA against NGFR or screener control were transfected with either p73β or vector control, followed by proliferation assay. (**E**) A model for NGFR regulation of p73. p73 transcriptionally activates NGFR, which in turn inactivates p73 by promoting its degradation via the CMA pathway.

Our new findings that NGFR promotes the degradation of p73 in cancer cells led us to question whether NGFR can also suppress p73-mediated cellular outcomes. We first tested this idea by employing IncuCyte ZOOM Live-Cell imaging system to monitor apoptosis in real time. H1299 cells were transfected with p73β alone or co-transfected with NGFR and incubated with fluorescein isothiocyanate-conjugated annexin V (FITC-annexin V). Transfection with NGFR alone served as a negative control. Fluorescent images were collected every 3 h over a span of 24 h. Quantification of annexin-V fluorescence demonstrates that apoptosis mediated by p73β was significantly attenuated in cells co-transfected with NGFR (Figure 6A). Representative images show the increased frequency of annexin V-positive cells in HCT116^p53−/−^ cells transfected with p73β indicating a higher level of apoptosis, but when co-transfected with NGFR, the degree of annexin-V fluorescence was comparable to controls (Supplementary Figure S5). Consistent with the data of cell apoptosis, cell proliferation assay shows an increased cell proliferation activity in H1299 cells when co-transfected with NGFR (Figure 6B). Furthermore, we performed colony formation assays in which we transfected H1299 cells with p73β alone or co-transfected with NGFR. Our results indicate that while p73β dramatically reduces the number of colonies formed as expected, co-transfection with NGFR significantly reduced p73β’s ability to inhibit clonogenicity (Figure 6C). To establish additional evidence that NGFR reduces p73 tumor suppressive activity, we utilized SK-MEL-147 cells with stable lentivirus expression of shNGFR or screener control, and overexpressed p73. Compared with control, cells with stable knockdown of NGFR demonstrated significantly enhanced susceptibility to p73β-mediated arrest in cellular proliferation (Figure 6D). These results show that NGFR suppresses p73-mediated induction of apoptosis as well as p73-mediated inhibition of clonogenicity in cancer cells.

## Discussion

The function of p73 as a tumor suppressor has been well established in *in vitro* and *in vivo* models (Flores et al., 2005; Rosenbluth and Pietenpol, 2008; Tomasini et al., 2008). Although p73 is rarely found to be mutated in cancers, several mechanisms have been recognized that it can suppress p73 activity in cancer cells, including hypermethylation of the TP73 promoter or amplification of proteins that inhibit TAp73 activity (Corn et al., 1999; Kawano et al., 1999; Casciano et al., 2002; Guan and Chen, 2005). Here, we identify NGFR as a novel p73 inactivator that serves as another example of an oncoprotein that can suppress p73 tumor suppressive activity. Indeed, NGFR has been shown to play a tumor supportive role in a variety of cancers, such as enhancing cancer cell survival in breast cancers, squamous cell carcinomas, melanomas, and schwannoma, and promote an invasive phenotype in gliomas, melanomas, and head and neck carcinomas (Descamps et al., 2001; Okumura et al., 2006; Soland et al., 2008; Truzzi et al., 2008; Boiko et al., 2010; Civenni et al., 2011; Kim et al., 2012). Our own studies have revealed the amplification of NGFR in gliomas (Zhou et al., 2016b), and our ongoing unpublished studies examining patient colorectal carcinoma (CRC) tumors indicate that not only NGFR is significantly higher expressed in CRC tumors compared with adjacent tissue, but NGFR is also associated with recurrent and metastatic CRC compared with non-recurrent and primary CRC. Our studies presented here unveil two novel findings: (i) a previously undiscovered mechanism of p73 degradation and (ii) a novel oncogenic function of NGFR through the inactivation of p73 by the CMA pathway.

We first demonstrate that NGFR is the transcriptional target of p73 target by overexpressing p73 in p53-null cancer cell lines HCT116^p53−/−^ and H1299 and performing RT-qPCR and WB analyses, and observed increased NGFR expression at both the mRNA and protein level (Supplementary Figure S2A–C). Furthermore, p73 stimulated the expression of a luciferase gene driven by the two responsive element (RE) sequences derived from the NGFR promoter (Supplementary Figure S2D). However, the p73 isoform lacking the transactivating domain, ΔNp73, was unable to induce NGFR expression (Supplementary Figure S2E). These data confirm, in cancer cells, the previous observation in mouse nervous system that NGFR is a *bona fide* p73 target gene (Niklison-Chirou et al., 2013). We then determined that NGFR in turn counteracts p73 function in a variety of cancer cells, as the ectopic overexpression of NGFR resulted in a decreased in p73 levels (Figure 1A and B), while shRNA-mediated knockdown of NGFR increased p73 levels (Figure 1C and D). In addition, p73 induction by chemotherapeutic reagents 5-FU and Eto was dramatically reduced in cells expressing ectopic NGFR expression (Figure 1E and F). We then demonstrate that ectopic NGFR expression reduces p73 transcriptional activity at both the mRNA (Figure 2A and B) and protein (Figure 2C) level and that this repression occurs independently of MDM2 (Figure 4C and D). Finally, we show that NGFR reduces the p73-driven luciferase activity of p21-Luc (Figure 2D). Repression of p73 function also manifested in both apoptosis assays and colony formation assays, as NGFR significantly reduced p73-mediated apoptosis and increased proliferation in cancer cells (Figure 6A and B), as well as reduced p73-mediated inhibition of clonogenicity (Figure 6C). Conversely, cells with shRNA-mediated stable knockdown of NGFR were significantly more susceptible to arrest in proliferation by p73β (Figure 6D). Collectively, these data designate NGFR as a negative feedback regulator of p73 tumor suppressive activity (Figure 6E).

To elucidate the mechanism by which NGFR inhibits p73 activity, we first detailed their interaction. Reciprocal co-IP assays indicated that they interact *in vitro* (Figure 3A and B) and independently of MDM2 (Figure 4A and B), as well as associate endogenously (Figure 3C), and further characterization revealed that NGFR binds to p73 DNA-binding domain, while p73 binds to NGFR N-terminal domain (Figure 3D and E). Interestingly, degradation of p73 by NGFR also occurred independently of MDM2 (Figure 4C), indicating that NGFR regulates p73 by a different mechanism than p53. Indeed, inhibition of proteasome function via MG132 did not affect the NGFR-mediated downregulation of p73 levels (Figure 5A). Surprisingly, the interaction between NGFR and p73 occurs primarily in the cytoplasm (Figure 5B). Analysis of the amino acid sequences of p73 and NGFR revealed KFERQ-like motifs that demarcate these two proteins as potential substrates for the CMA pathway (Figure 5C). As such, whereas the inhibition of proteasome had no effect, inhibition of lysosome activity rescued NGFR-mediated degradation of p73 (Figure 5D and E) and serum starvation enhanced this degradation (Figure 5F). Furthermore, knocking down the key receptor in CMA, LAMP2a, prevented NGFR from downregulating p73 (Figure 5G). Finally, co-IP assays show that p73 and NGFR interact with the key mediators of the CMA (Figure 5H and I). Altogether, our data demonstrate that overexpression of NGFR in tumors may serve as a mechanism by which cancer cells hijack the negative feedback regulation of p73 by NGFR for selective growth advantage. Interestingly, the oncoprotein isoform of p73, ΔNp73, was unable to induce NGFR expression, indicating no feedback loop exists between ΔNp73 and NGFR (Supplementary Figure S2E). We further show that p73 interacts with CMA mediators LAMP2a and HSPA8 via its N-terminus (Supplementary Figure S4), suggesting that NGFR regulating ΔNp73 via the CMA is unlikely and that NGFR-mediated anti-p73 activity is limited to TAp73.

The CMA differs from macroautophagy; while macrophagy is principally a non-selective event in which cytosolic components are enveloped into a double-membrane autophagosome prior to fusion with lysosome, the CMA targets specific proteins that contain a recognition motif to be delivered and degraded directly by the lysosome (Cuervo and Wong, 2014). General physiologic functions of the CMA include energy conservation during the starvation and cellular quality control by removing damaged proteins, particularly under conditions of oxidative stress (Cuervo et al., 1995; Kiffin et al., 2004). More cell-specific roles for CMA include kidney growth, neuronal survival, and antigen presentation (Franch et al., 2001; Zhou et al., 2005; Yang et al., 2009). In cancers, increased basal CMA activity and elevated LAMP2a expression in different cancer types are observed and associated with cancer cell proliferation, tumor growth, metastasis, removal of tumor suppressors, and promoting metabolic switch favorable for cell growth (Kon et al., 2011; Saha, 2012; Quintavalle et al., 2014; Zhang et al., 2014; Zhou et al., 2016a). Our discovery that NGFR promotes p73 degradation through the CMA not only uncovers a new mechanism by which p73 can be downregulated but also identifies NGFR as a regulator of the CMA, serving as a co-chaperone to bind to p73 and deliver it to the lysosome for targeted proteolysis (Figure 6E). Our findings are novel in the sense that NGFR typically functions as a transmembrane pan-receptor involved in the initiation, development, and maintenance of the nervous system and human cancers and instead connects NGFR with the CMA, another cancer-associated pathway. Our study therefore uncovers a new receptor-independent, intracellular, and tumor-promoting function for NGFR.

## Materials and methods

### Cell culture and transient transfection

H1299, HCT116^−/−^, U-373, U-118, and MEF^p53−/−;MDM2−/−^ cells were cultured in Dulbecco’s modified Eagle’s medium supplemented with 10% fetal bovine serum, 50 U/ml penicillin, and 0.1 mg/ml streptomycin. All cells were maintained at 37°C in 5% CO_2_ humidified atmosphere. Cells were transfected with plasmids as indicated in figures using TurboFect reagent following the manufacturer’s instruction (Thermo Scientific).

### Plasmids and antibodies

The Myc-tagged NGFR expression plasmid was generated by inserting the full-length cDNA amplified by PCR from the previously purchased pDSRed-NGFR (Addgene, Dr Moses Chao) into the pcDNA3.1/Myc-His vector, using the following primers: 5′-CCGGAATTCATGGGGGCAGGTGCCACC-3′ and 5′-CGCGGATCCCACCGGGGATGTGGCAGT-3′. The pGL3-RE1 and pGL3-RE2 plasmids were generated by inserting the genomic DNA covering p53 RE1 or RE2 into the pGL3-promoter vector using the following primers: 5′-CGGGGTACCTTCTACTGTCATGTCAAAGGAA-3′ and 5′-CCGCTCGAGCCCTCCAGCTACTACTCAGAC-3′ for RE1; 5′-CGGGGTACCGGCAAGTGGCATTGGTGGTA-3′ and 5′-CCGCTCGAGTCGTTTGTAAAGTGGGCATAA-3′ for RE2. The Flag-tagged TAp73β expression plasmid was generated by inserting the full-length cDNA amplified by PCR from TAp73β vector into the pcDNA3-2Flag vector using the following primers, 5′-CGCGGATCCATGGCCGAGTCCACCGC-3′ and 5′-CCGCTCGAGTCAGGGCCCCCAGGTCCT-3′. The lentiviral-based plasmid NGFR shRNA-1 was generated by inserting the sequence 5′-CCGGCCGAGCACATAACTCCTTTACTCGAGTAAAGGAGTCTATGTGCTCGGTTTTTG-3′ into pLKO.1 vector. The plasmid for NGFR shRNA-2 was purchased (Sigma-Aldrich). The plasmids encoding Flag-MDM2, Flag-TAp63, and WWP-Luc (p21/WAF1 promoter) were described previously (el-Deiry et al., 1993; Dai et al., 2004; Liao et al., 2013). Anti-Flag (Sigma-Aldrich), anti-p73 (E-4, Santa Cruz Biotechnology), anti-NGFR (D4B3, Cell signaling Technology), anti-p21 (CP74, Neomarkers), anti-HSPA8 (B-6, Santa Cruz Biotechnology), anti-Lamp2a (H4B4, Santa Cruz Biotechnology), anti-β-actin (C4, Santa Cruz Biotechnology), and anti-GAPDH (Millipore) were commercially purchased.

### Reverse transcription and quantitative PCR analysis

Total RNA was isolated from cells using Trizol (Invitrogen) following the manufacturer’s protocol. Total RNAs of 0.5–1 μg were used as templates for reverse transcription using poly-(T)20 primers and M-MLV reverse transcriptase (Promega). Quantitative PCR (qPCR) was conducted using SYBR Green Mix according to the manufacturer’s protocol (BioRad). The following primers were used: *NGFR*, 5′-CCTGGACAGCGTGACGTTC-3′ and 5′-CCCAGTCGTCTCATCCTGGT-3′; *p21*, 5′-CTGGACTGTTTTCTCTCGGCTC-3′ and 5′-TGTATATTCAGCATTGTGGGAGGA-3′; *MDM2*, 5′-ATGAATCCCCCCCTTCCAT-3′ and 5′-CAGGAAGCCAATTCTCACGAA-3′; mouse *p21*, 5′-CCAGCAGAATAAAAGGTGCCACAGG-3′ and 5′-GCATCGCAATCACGGCGCAA-3′.

### IB and IP

Cells were lysed in lysis buffer consisting of 50 mM Tris/HCl (pH 7.5), 0.5% Nonidet P-40 (NP-40), 1 mM EDTA, 150 mM NaCl, 1 mM phenylmethylsulfonyl fluoride, 0.25 mg/ml pepstatin A, and 1 mM leupeptin. Equal amounts of clear cell lysates (20–50 μg protein) were used for IB analysis. IP was conducted using antibodies as indicated in figures for 3–4 h, followed by addition of IgG or IgA beads for 1–2 h. These beads were washed three times with lysis buffer, and bound proteins were detected by IB.

### Luciferase reporter assay

H1299 cells were transfected with Renilla-Luc together with the specified plasmids in figure legends. Forty-eight hours post-transfection, firefly luciferase activities were measured and normalized by a factor of Renilla luciferase activity in the same assay using the Dual-Glo Luciferase Assay System (Promega).

### GST fusion protein association assay

GST-tagged p73 fragments were expressed in *E. coli* and conjugated with glutathione-Sepharose 4B beads (Sigma-Aldrich). Protein–protein interaction assays were conducted by using cell lysates with mammalian-expressed Myc-NGFR, HSPA8, or LAMP2A. Briefly, the cell lysates were incubated and gently rotated with the glutathione-Sepharose 4B beads containing 500 ng of GST-p73 fragments or GST only at 4°C for 4 h. The mixtures were washed three times with GST lysis buffer (50 mM Tris/HCl, pH 8.0, 0.5% NP-40, 1 mM EDTA, 150 mM NaCl, and 10% glycerol). Bound proteins were analyzed by IB with the antibodies as indicated in figures.

### RNA interference

The siRNAs against LAMP2a were commercially purchased from Ambion, and 40 nM siRNA was introduced into cells using TurboFect according to manufacturer’s protocol. Thirty-six hours later, cells were transfected with NGFR for 36 h, followed by IB.

### Cell apoptosis and proliferation assays

IncuCyte S3 Live-Cell Analysis System (Essen Bioscience) was used for kinetic monitoring of apoptosis and proliferation. Transiently transfected H1299 cells were plated into 96-well plates at a density of 1500 cells per well. For apoptosis assay, cells were incubated with Annexin V Green (Essen Bioscience), which is conjugated with fluorescein isothiocyanate and emits a photostable fluorescent signal when bound to phosphatidylserine. Plates were scanned and fluorescent or phase-contrast images were captured in real time for every 3 h over a span of 24 or 60 h. Data were analyzed by the IncuCyte S3 Basic Analyzer software module.

### Colony formation assay

Cells were transfected with different plasmids totally to equivalent amounts. The next day, 3000 cells per experimental group were seeded into 60-mm plates. Geneticin was added to the medium and the medium was changed every 3 days until colonies were visible. Cells were then fixed with methanol and stained with 0.5% crystal violet solution at room temperature for 30 min. ImageJ was used for the quantification of colonies.

### Statistical analysis

The Student’s two-tailed *t*-test was used to determine the mean differences between treatment and control. Data are presented as mean ± SEM.

## Supplementary Material

mjaa017_Supplementary_material
