# Supplementary material for: Inhibition of tumor suppressor p73 by nerve growth factor receptor via chaperone-mediated autophagy
Source: J Mol Cell Biol. 2020 Apr 13;12(9):700–12. doi: 10.1093/jmcb/mjaa017 (PMC7749740; doi:10.1093/jmcb/mjaa017)
Supplement: mjaa017_Supplementary_material [file mjaa017_supplementary_material.pdf]

Supplementary material

Inhibition of tumor suppressor p73 by nerve growth factor receptor via chaperone-mediated autophagy

Daniel Nguyen[1], Kun Yang[1], Lucia Chao[1,3], Yun Deng[1,4], Xiang Zhou[1,5], Zhen Zhang[2], Shelya X. Zeng[1], and Hua Lu[1,*]

[1] Department of Biochemistry and Molecular Biology, Tulane Cancer Center, Tulane University School of Medicine, New Orleans, LA 70112, USA

[2] Department of Radiation Oncology, Shanghai Cancer Center, and Department of Oncology, Shanghai Medical School, Fudan University, Shanghai 200032, China

[3] Present address: Verna and Marrs McLean Department of Biochemistry and Molecular Biology, Baylor College of Medicine, Houston, Texas 77030, USA

[4] Present address: Department of Radiation Oncology, Shanghai Cancer Center, Fudan University, Shanghai 200032, China

[5] Present address: Institute of Biomedical Sciences, Shanghai Cancer Center, Fudan University, Shanghai 200032, China

*Correspondence to: Hua Lu, Tel: (504) 988-5293; E-mail: hlu2@tulane.edu

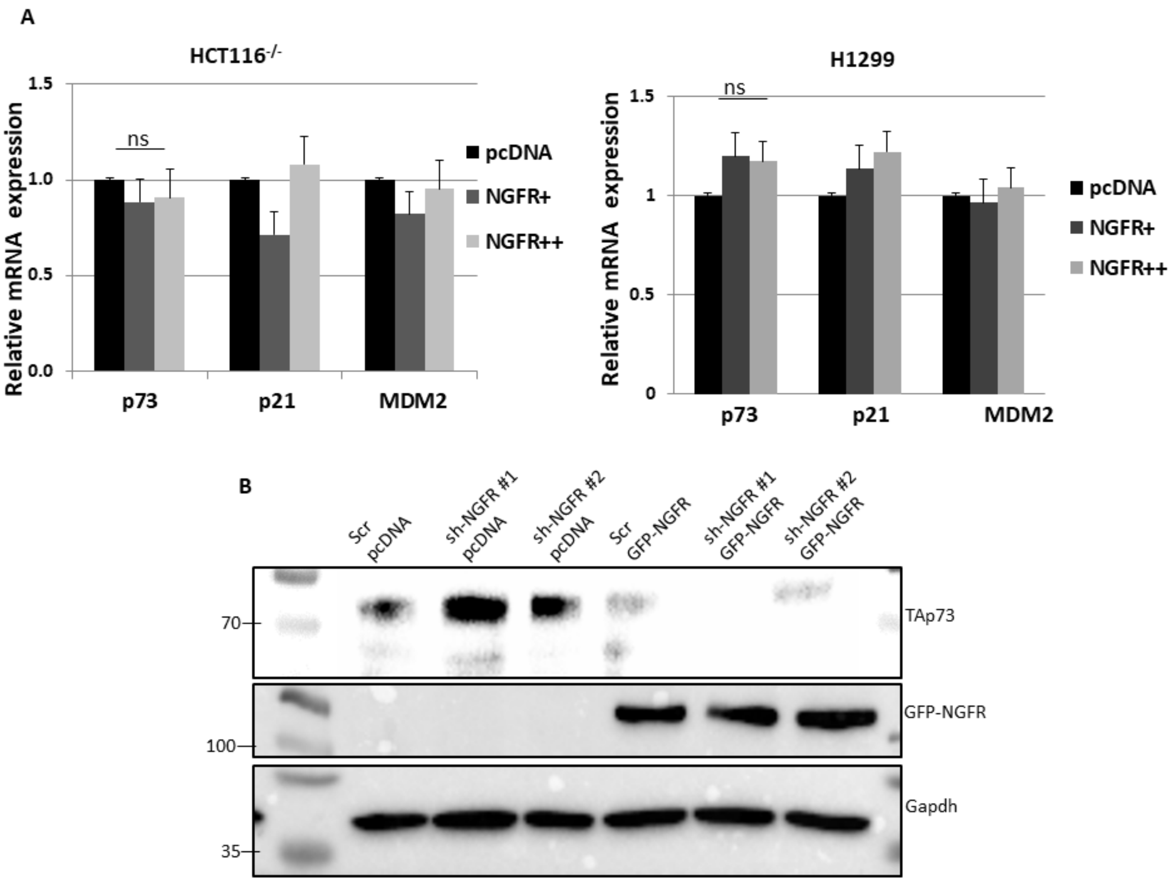

Supplementary Figure S1

(**A**) **NGFR does not reduce p73 mRNA levels.** HCT116$^{-/-}$ and H1299 cells were transfected with different dosages of MYC-NGFR for 30h and p73, p21, and MDM2 expression was determined by q-PCR. Three biological replicates were used for *P*-value, **P*<0.05.

(**B**) **Rescue of shRNA-mediated elevation of endogenous p73 levels.** H1299 cells were transfected with lentiviral-based plasmids expressing shRNA targeting two different NGFR sequences of control shRNA for 48h, followed by transfection with GFP-NGFR for 36h and IB with the indicated antibodies.

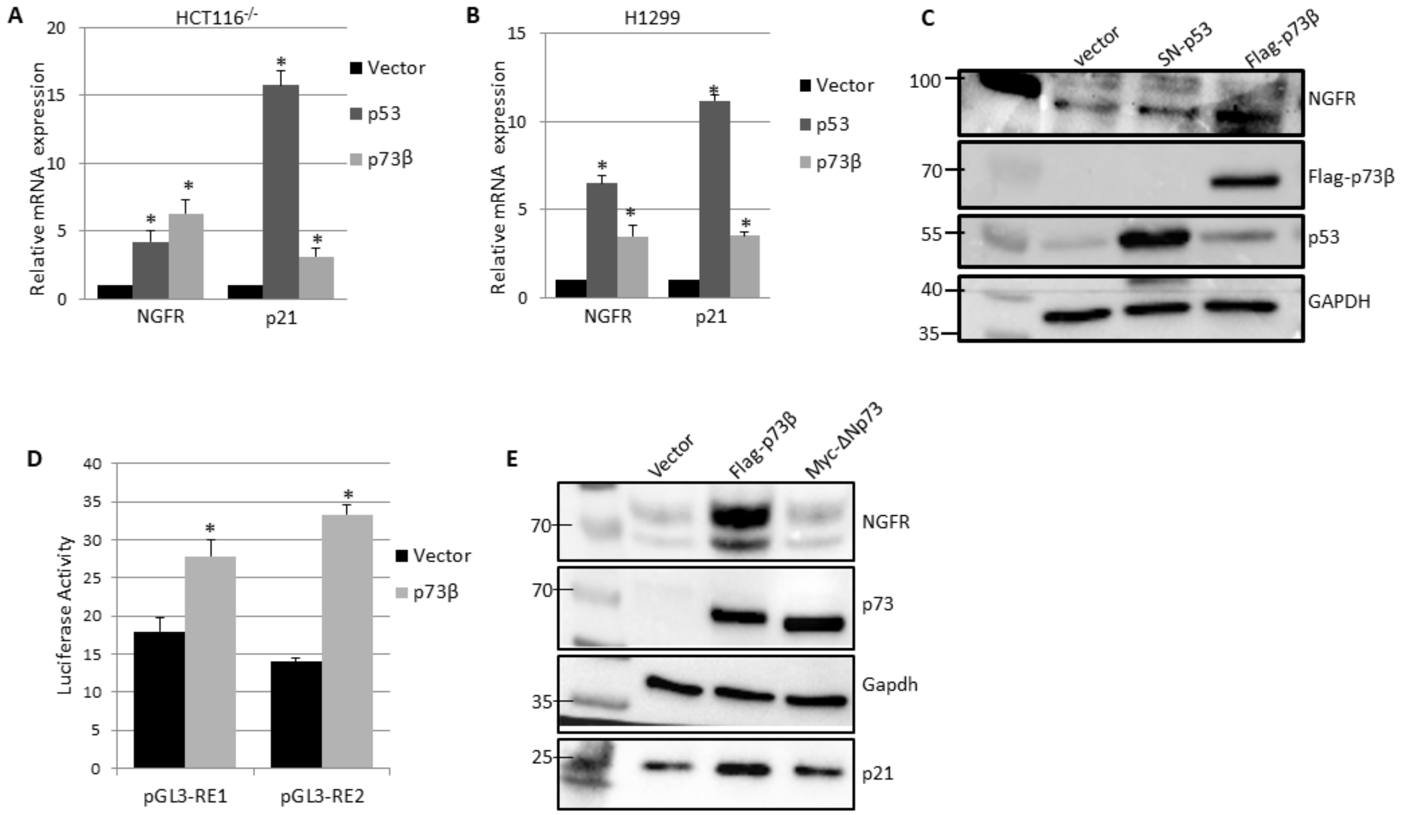

Supplementary Figure S2

p73 transcriptionally activates NGFR expression in human cancer cells. (**A** and **B**) NGFR mRNA is elevated by ectopic p73β expression in p53-null cells. HCT116P53$^{-/-}$ (**A**) and H1299 (**B**) cells were transfected with p53, p73β, or vector control for 30 h and NGFR and p21 expression was determined by q-PCR. Three biological replicates were used for *P*-value, **P*<0.05. (**C**) NGFR protein levels are increased by ectopic p73β expression. HCT116P53$^{-/-}$ cells were transfected with p53, p73β, or vector control for 48 h followed by immunoblot (IB) with the indicated antibodies. (**D**) p73β induces luciferase activity through both RE1 and RE2. H1299 cells were transfected with plasmids expressing two potential response elements (RE1 and RE2) linked to luciferase reporter gene, pCMV-β-galactoside (β-gal), and either p73β or control vector. Forty-eight hours after transfection, firefly luciferase activities were determined and normalized by a factor of β-gal activity in the same assay. Three biological replicates were used for *P*-value, **P*<0.05. (**E**) Full length p73, but not ΔNp73, can induce expression of endogenous NGFR. H1299 cells were transfected with either p73β, ΔNp73, or vector control for 48 h followed by IB with the indicated antibodies.

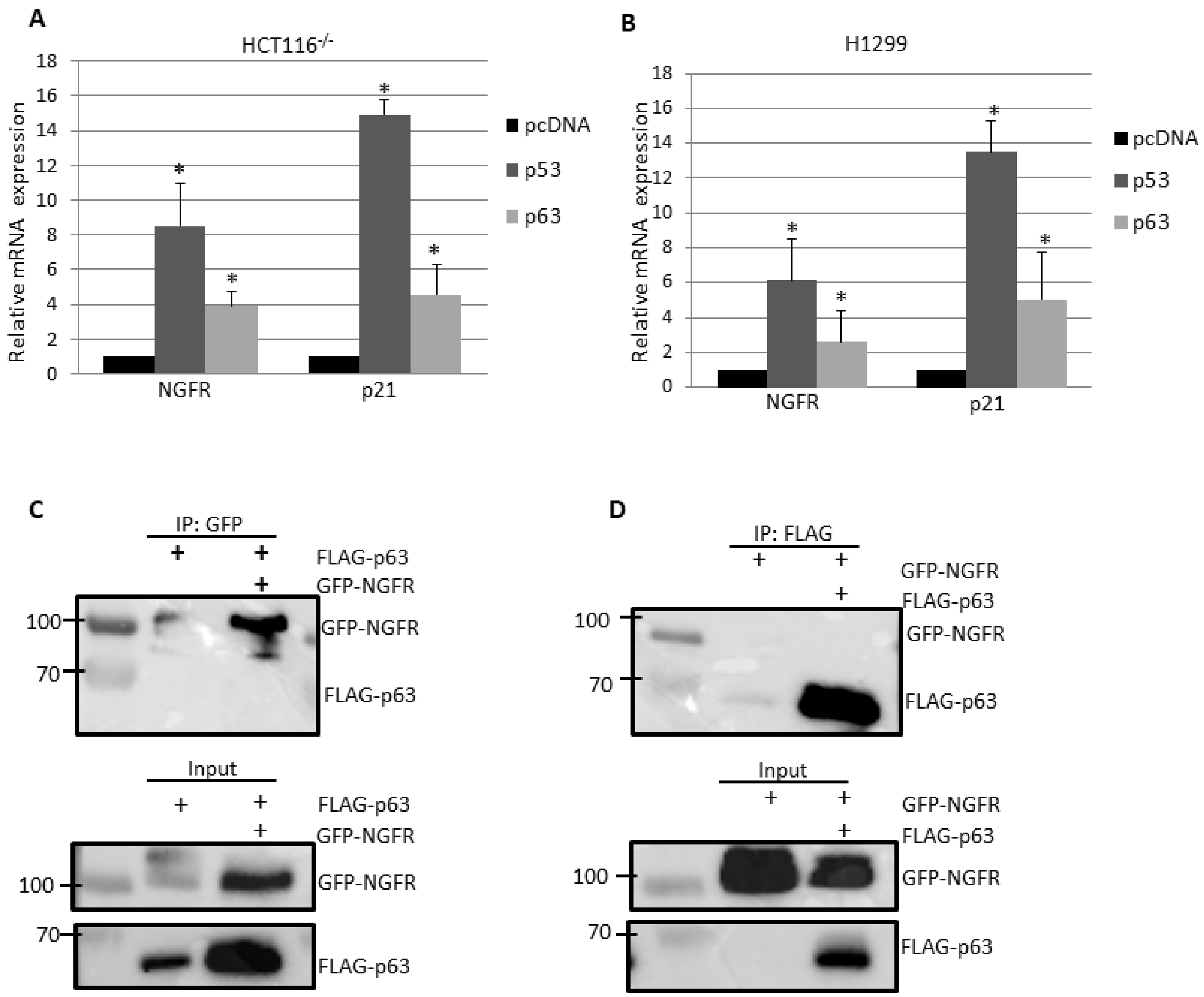

Supplementary Figure S3

p63 induces NGFR expression, but NGFR does not bind to p63. (**A** and **B**) NGFR mRNA is elevated by ectopic p63 expression in p53-null cells. HCT116P53$^{-/-}$ (**A**) and H1299 (**B**) cells were transfected with p63, p53, or vector control for 30 h and NGFR and p21 expression was determined by q-PCR. Three biological replicates were used for *P*-value, **P*<0.05. (**C** and **D**) p63 and NGFR do not interact. H1299 cells were transfected with plasmids encoding Flag-p63, GFP-NGFR, or both, followed by co-immunoprecipitation (co-IP) and immunblotted with the indicated antibodies.

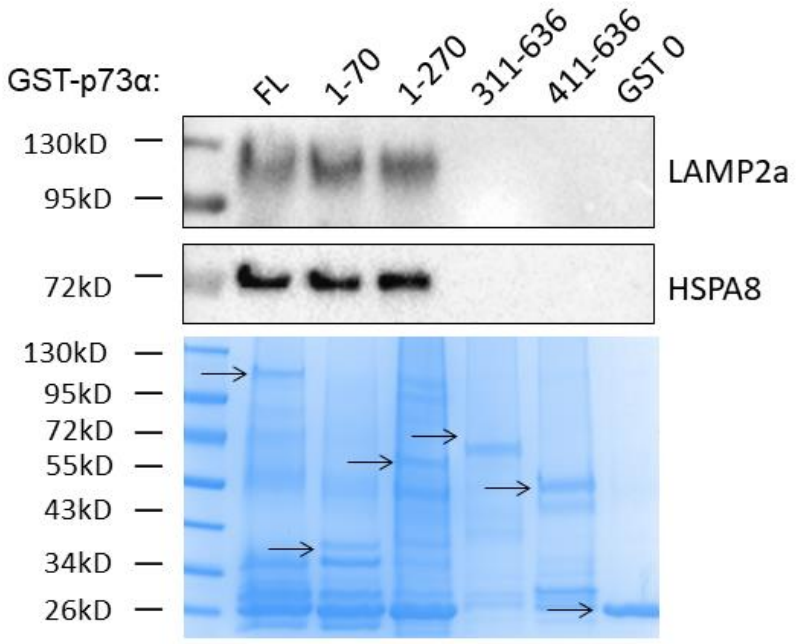

Supplementary Figure S4

Mapping the HSPA8 and LAMP2A binding domain of p73α by GST-pull down assays. H1299 cells were treated with 1% serum starvation for 8 h and cell lysate was incubated with purified GST-tagged p73α full length and fragments, including aa 1–70, aa 1–270, aa 311–636, aa 411–636, and GST protein alone for one hour at room temperature. Bound proteins were detected by immunoblotting using anti-HSPA8, anti-LAMP2A antibodies or coomassie staining.

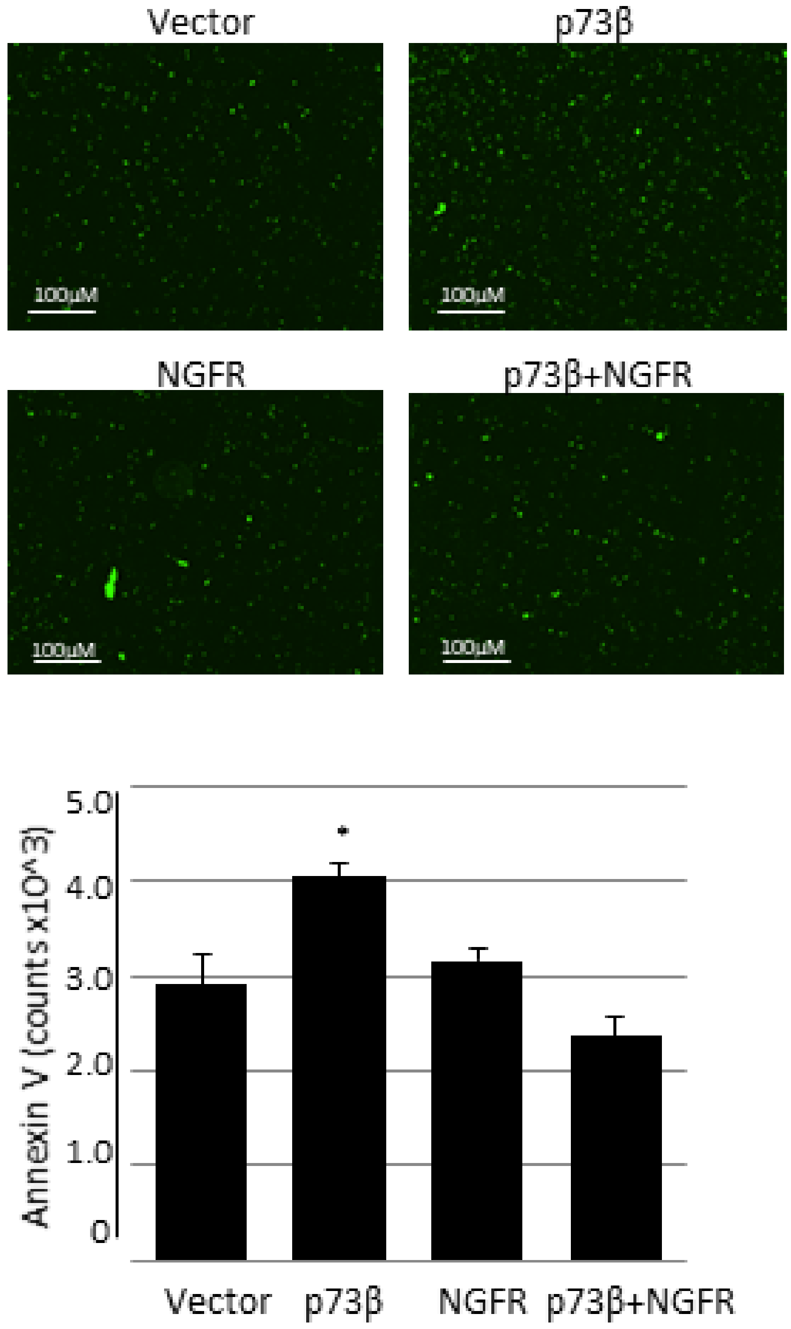

Supplementary Figure S5

NGFR attenuates p73β mediated apoptosis. HCT116$^{-/-}$ cells transfected with indicated plasmids and cell apoptosis were measured by IncuCyte® S3 Live Cell Analysis System. Representative images show the frequency of annexin V-positive cells.
